# The electrophysiological footprint of *CACNA1A* disorders

**DOI:** 10.1007/s00415-021-10415-x

**Published:** 2021-02-05

**Authors:** Elisabetta Indelicato, Iris Unterberger, Wolfgang Nachbauer, Andreas Eigentler, Matthias Amprosi, Fiona Zeiner, Edda Haberlandt, Manuela Kaml, Elke Gizewski, Sylvia Boesch

**Affiliations:** 1 Center for Rare Movement Disorders Innsbruck, Department of Neurology, Medical University of Innsbruck, Anichstrasse 35, 6020 Innsbruck, Austria; 2 Epileptology Division, Department of Neurology, Medical University of Innsbruck, Innsbruck, Austria; 3 Department of Pediatrics I, Medical University of Innsbruck, Innsbruck, Austria; 4 Department of Pediatrics, City Hospital, Dornbirn, Austria; 5 Department of Neuroradiology, Medical University of Innsbruck, Innsbruck, Austria

**Keywords:** Voltage-gated calcium channels, Familial hemiplegic migraine type 1, Episodic ataxia type 2, Spinocerebellar ataxia type 6, Epilepsy, Intermittent epileptic discharges

## Abstract

**Objectives:**

CACNA1A variants underlie three neurological disorders: familial hemiplegic migraine type 1 (FHM1), episodic ataxia type 2 (EA2) and spinocerebellar ataxia type 6 (SCA6). EEG is applied to study their episodic manifestations, but findings in the intervals did not gain attention up to date.

**Methods:**

We analyzed repeated EEG recordings performed between 1994 and 2019 in a large cohort of genetically confirmed CACNA1A patients. EEG findings were compared with those of CACNA1A-negative phenocopies. A review of the related literature was performed.

**Results:**

85 EEG recordings from 38 patients (19 EA2, 14 FHM1, 5 SCA6) were analyzed. Baseline EEG was abnormal in 55% of cases (12 EA2, 9 FHM1). The most common finding was a lateralized intermittent slowing, mainly affecting the temporal region. Slowing was more pronounced after a recent attack but was consistently detected in the majority of patients also during the follow-up. Interictal epileptic discharges (IEDs) were detected in eight patients (7 EA2,1 FHM1). EEG abnormalities and especially IEDs were significantly associated with younger age at examination (16 ± 9 vs 43 ± 21 years in those without epileptic changes, *p* = 0.003) and with earlier onset of disease (1 (1–2) vs 12 (5–45) years, *p* = 0.0009). EEG findings in CACNA1A-negative phenocopies (*n* = 15) were largely unremarkable (*p* = 0.03 in the comparison with CACNA1A patients).

**Conclusions:**

EEG abnormalities between attacks are highly prevalent in episodic CACNA1A disorders and especially associated with younger age at examination and earlier disease onset. Our findings underpin an age-dependent effect of CACNA1A variants, with a more severe impairment when P/Q channel dysfunction manifests early in life.

## Introduction

The gene *CACNA1A* encodes the pore-forming α1A subunit of the neuronal channel P/Q, which belongs to the superfamily of voltage-gated calcium channels [1]. P/Q channels are ubiquitous in the central nervous system and particularly abundant in cerebellar granules and Purkinje cells. P/Q channels play a pivotal role in neurotransmission due to their localization at the presynaptic terminals where they ensure the calcium-induced vesicular exocytosis of neurotransmitter. The selective biological functions of the voltage-gated calcium channels are determined by their specific α1 subunits. Indeed, the α1 subunit defines the channel-gating property and thus represents the main modulator of calcium currents. In this light, it is not surprising that *CACNA1A* gene is especially nontolerant to functional variants [2].

Ion channel variants typically underlie disorders with episodic manifestations. Accordingly, pathogenic *CACNA1A* variants have been initially identified in the setting of familial hemiplegic migraine type 1 (FHM1) and episodic ataxia type 2 (EA2) [3]. FHM1 is a monogenic migraine form characterized by motor deficits during the aura and is typically associated with gain-of-function *CACNA1A* variants [3]. EA2 manifests with self-limiting attacks of ataxia with dizziness, ocular disturbances, and dysarthria, and is generally caused by nonsense mutations or large rearrangements resulting in a loss-of-function [3]. Both disorders usually present in the first 2 decades and may display various degrees of phenotypic overlap. Beyond paroxysmal manifestations, mild to moderate chronic cerebellar signs can develop in the course of the disease in up to 75% of patients with FHM1 and EA2 [4, 5]. Interestingly, a late-onset chronic disorder, the spinocerebellar ataxia type 6 (SCA6), has been also linked to *CACNA1A* variants. SCA6 is underlined by an expanded polyglutamine tract in the C-terminal of the gene, a modification which does not primarily affect calcium currents [6, 7]. SCA6 shares with other spinocerebellar ataxias a similar phenotype with chronic progressive cerebellar dysfunction. Nevertheless, paroxysmal symptoms often occur at disease onset in SCA6 [8], resembling those of its allelic episodic disorders.

In the clinical approach to paroxysmal neurological disorders, electroencephalogram (EEG) is an essential part of the initial diagnostic workup. In the present study, we analyzed routine scalp EEGs (rsEEG) of a large cohort of genetically confirmed *CACNA1A* patients referring to the Center for Rare Movement Disorders at the Medical University of Innsbruck. Furthermore, we performed a comparison with EEG findings from *CACNA1A* phenocopies referring to our center and reviewed the current related literature on the topic. We aimed at defining the EEG pattern of *CACNA1A*-related disorders to support clinical evaluation and shed further light on the phenomenology resulting from P/Q channel dysfunction.

## Methods

### Patients

Patients with genetically confirmed *CACNA1A* disorders were retrieved from the database of the Center for Rare Movement Disorders at the Department of Neurology of the Medical University of Innsbruck. Additionally, *CACNA1A*-negative phenocopies were considered as the control group. Across the manuscript, families are labeled with Arabic numbers and family members with Roman numbers. Basic clinical and demographic data as well as findings from neurological examination were collected. Brain MR imaging was performed within the routine workup either at a 1.5 or 3 T scanner.

### EEG evaluation

*CACNA1A* patients underwent rsEEG examinations within their regular clinical follow-up. We retrieved 85 rsEEG recordings which were performed between 1994 and 2019 either at the Department of Neurology or at the Department of Pediatrics of our university hospital, including the recordings from video EEG monitoring with scalp electrodes. All rsEEGs were recorded during wakefulness. EEG terminology report and interpretation were classified according to the definition by Kane et al. [9]. The following EEG findings were defined as abnormal EEG patterns: (a) abnormal changes in normal rhythm, (b) abnormal slow wave activity including theta- and/or delta-frequencies, and (c) interictal epileptiform discharges (IEDs). Each of these findings may be altered alone or in combination, may be localized (i.e., in one or two lobes), lateralized, bilateral or generalized and may occur continuously or intermittently.

EEG findings were analyzed by EI and reviewed by a senior neurologist with long-time experience in EEG evaluation (IU, MK, FZ or EH). Senior physicians were blinded to clinical diagnosis and medical history at the time of EEG.

### Statistical analysis

We performed statistical analyses using SPSS version 25. Data are reported as mean ± SD or median (interquartile range) according to their distribution, which was tested by means of Kolmogorov–Smirnov test. Intergroup comparisons were performed by means of *t *test, Mann–Whitney-*U* and chi-squared test depending on the variable category. Statistical significance was set at *p* < 0.05.

### Review of the literature

A Medline search was conducted applying the following keywords: *CACNA1A*, hemiplegic migraine, episodic ataxia, epilepsy, and electroencephalogram/EEG. Relevant references from the retrieved papers were also selected. We considered only the report of genetically confirmed *CACNA1A* cases.

## Results

### Innsbruck *CACNA1A* cohort: clinical history and neurological findings

Fifty-three genetically confirmed *CACNA1A* patients were registered in our database. We considered for the present study 38 patients (13 women, 25 men) who underwent rsEEG examination at least once. Twenty-five subjects have already been described in the previous publications of our group [10, 11]; 13 patients and 4 new *CACNA1A* variants are described herein for the first time. Clinical and demographic data are summarized in Table 1.

**Table 1:** Clinical features of Innsbruck *CACNA1A* patients who underwent EEG examination

Family	Genotype	Phenotype	Pt. ID	Age at onset	Age at first EEG	Chronic cerebellar signs	Cerebellar atrophy at MRI	Pathologic findings at first EEG	IEDs^a^
1	R1667W	FHM1	1-I*	12	56	x	x	x	
			1-II	34	42	x	x	x	
			1-III	58	58	x	x	x	
			1-IV	1	11	x	x	x	x
2	c.3102 + 2 T > C	EA2	2-I	1	28	x	x	x	x
			2-II*	27	37	x	x	x	
3	T666M	FHM1	3-I	40	71	x	x		
			3-II	5	47	x	x	x	
			3-III	13	42	x	x		
			3-IV	4	18	x	x		
			3-V	7	10	x	x	x	
			3-VI	1,5	2		na		
			3-VII*	11	11		x	x	
			3-VIII*	1,5	3	x	na		
4	c.3089 + 2 T > C	EA2	4-I	10	54	x	x		
			4-II	45	54	x	x	x	
			4-III	2	15	x		x	x
			4-IV	10	20	x	X		x
			4-V	1	26	x		x	x
			4-VI*	1	3		na	x	x
5	R198Q	EA2	5-I	1	22	x	x	x	x
6	S218L	FHM1	6-I	1	6	x	x	x	
7	c.959G > A	EA2	7-I	8	48	x	x		
8	c.3603dup	EA2	8-I	7	45	x	x		
9	G540R	EA2	9-I	1,5	18		x	x	
10	C1869R	EA2	10-I*	36	41	x	x	x	
11	I239T**	EA2	11-I*	61	68	x	x		
			11-II*	55	73	x	x		
12	R2248H**	EA2	12-I*	45	41	x	x	x	
13	D2173Y**	EA2	13-I*	50	52	x	x	x	
14	T666M	FHM1	14-I*	2	41	x	x	x	
15	A754V**	EA2	15-I*	1	1				x
16	Q1154X	EA2	16-I*	3	64	x	x	x	
17	12/23	SCA6	17-I	55	67	x	x		
	13/23		17-II	40	62	x	x		
	14/23		17-III	47	48	x	x		
18		SCA6	18-I	63	67	x	x		
19	8/23	SCA6	19-I	36	39	x	x		

IEDs: interictal epileptic discharges

*These patients have been described herein for the first time

**Newly reported *CACNA1A* variants

^a^Both first EEG and follow-up EEGs are considered

Our *CACNA1A* cohort comprehended 14 patients from 4 FHM1 families, 19 patients from 12 EA2 families, and 5 patients from 3 SCA6 families. Mean age at examination was 37 ± 22 years (range 1–73 years). Episodic symptoms were reported by 25 patients at the time of referral (66%) and 7 other patients (18%) had experienced episodic symptoms at the beginning of the disease. Thirty-three patients (87%) showed chronic cerebellar signs at the neurological examination.

Brain MR imaging was available for all patients but two. In 35 cases (92%), cerebellar atrophy was evident. The degree of cerebellar atrophy was rather mild in the majority of FHM1 and EA2 patients as well as mostly limited to the vermis.

### First EEG findings

A majority of patients were drug-naive at the time of the first rsEEG (92%, *n* = 34). Of the remaining 4, *n* = 3 were on acetazolamide and *n* = 1 on flunarizine. Seven patients (18%) had experienced attacks in the week preceding the examination.

Overall, pathological EEG findings were detected in 21 patients (55%; 11 FHM1, 10 EA2). Clue pathologic findings consisted of an intermittent slowing in delta–theta-frequency range (*n* = 19, 90%). In one further patient, EEG displayed a continuous left lateralized hemispheric slowing indicative of a recent attack. Delta–theta activity was lateralized in five cases (*n* = 3 left hemisphere, *n* = 2 right hemisphere), bilateral in nine cases (of which four with more pronounced slowing in the left hemisphere) and generalized in seven cases. In the majority of the cases, delta–theta activity involved more markedly or selectively the temporal region (*n* = 13, 62%). Three patients (6%) had IEDs in their first EEG (see Tables 2 and 3 for details).

**Table 2:** Synopsis of pathological findings at first rsEEG

Pt. ID	Phenotype	Age	Background rhythm	Intermittent slowing (frequency range)	Continuous slowing (frequency range)	Bilateral or generalized	Lateralized (R/L)	Regional (F/C/T/P/O)	IEDs
1-I	FHM1	56	A	T/D		Bilateral		FT	
1-II	FHM1	42	A	D/T		Non lateralized/bilateral	L > R	T	
1-III	FHM1	58	A	D/T			L		
1-IV	FHM1	11	A	T		Bilateral		FT	
2-I	EA2	28	A	T/D		Generalized			
2-II	EA2	37	A	D/T		Generalized			
3-II	FHM1	47	A	T/D		Bilateral		T	
3-V	FHM1	10	A	T/D			L	TP	
3-VII	FHM1	11	A	T/D			L	T	
4-II	EA2	54	A	T		Generalized			
4-III	EA2	15	A	T/D		Generalized			yes
4-V	EA2	26	A	D/T		Non lateralized/bilateral	L > R	T	yes
4-VI	EA2	3	T			Generalized			yes
5-I	EA2	22	A	T		Bilateral		T	
6-I	FHM1	6	D		D		R	FT	
9-I	EA2	18	A	T		Bilateral		T	
10-I	EA2	41	A	D/T		Generalized			
12-I	EA2	41	A	T		Generalized			
13-I	EA2	52	A	D/T		Non lateralized/bilateral	L > R	T	
14-I	FHM1	41	A	T			R	T	
16-I	EA2	64	A	T/D		Non lateralized/bilateral	L > R	T	

Concerning frequencies: *A* alpha, *T* theta, *D* delta; concerning localization: L and R = left and right hemispheres respectively, F = frontal, C = central, T = temporal, P = parietal, and O = occipital regions. IEDs: intermittent epileptic discharges. When both T and D frequencies were detected, the one that recurred most in EEG is reported after the slash. The slowing can occur continuously or intermittently; it can be generalized, bilateral or lateralized. In case of bilateral slowing, the presence of a side difference is also reported (for example L > R). Bilateral or lateralized slowing can also affect specifically one or two lobes (regional slowing)

**Table 3:** Interictal epileptic discharges (IEDs) in *CACNA1A* patients

Pt. ID	Phenotype	Age at onset	Age at EEG	IEDs
1-IV	FHM1	1	11	Bilateral frontal sharp waves
2-I	EA2	1	Childhood*	Generalized spike waves
4-III	EA2	2	15	Generalized spike wave complexes
4-IV	EA2	10	20	Superimposed spikes in generalized rhythmic delta
4-V	EA2	1	26	Left temporal sharp waves
4-VI	EA2	1	3	Superimposed spikes in generalized rhythmic delta
5-I	EA2	1	30	Left temporal sharp waves
15-I	EA2	1	6	Superimposed spikes in generalized rhythmic delta

*The first rsEEG at our department was performed at 28 years of age. IEDs were documented in previous EEGs

Analyzing *CACNA1A* subgroups, all five SCA6 patients had normal EEG, while FHM1 and EA2 patients had a similar frequency of unspecific slowing. IEDs at the first EEG were detected only in EA2 patients. Although EEG findings were more severe in patients with recent attacks as compared to patients without recent attacks, this difference was not statistically significant (*p* = 0.2). Interestingly, age at examination was lower in patients with pathological EEG findings (32 ± 19 vs 43 ± 25 years in those with normal EEG, *p* = 0.1, non-significant). Moreover, patients with pathological EEG findings had an earlier onset of symptoms as compared to those with normal EEGs (16 ± 19 years of age at onset vs 26 ± 25, *p* = 0.1, non-significant).

### EEG findings during an attack

Three EA2 patients (Pt. 4-V, 9-I, 13-I) were admitted to the hospital during an attack because of severe neurological impairment and underwent in-patient prolonged video-EEG monitoring. In this setting, several habitual attacks were documented. During clinically observed attacks EEG was unremarkable. Four FHM1 patients underwent EEG recordings during migraine attacks (Pt. 3-I, 3-IV, 3-V, 6-I). In these cases, a continuous delta activity was documented over the hemisphere contralateral to their aura symptoms.

### Follow-up EEG findings

Eighteen patients underwent repeated rsEEG examinations during the disease course.

Short-term follow-up in two patients (Pt. 3-VII and 9), 1–2 months after the first rsEEG, which in both cases was performed after an attack, showed unchanged slowing. Another short-time rsEEG follow-up after starting acetazolamide remained without improvement as compared to the first rsEEG (Pt. 2-II).

In the remaining patients repeated rsEEG examinations were performed during a mean follow-up of 9 ± 5 years. In five patients (36%) the findings remained unchanged. Other five patients (36%) displayed a worsening consisting of appearing of slowing, whereas the first rsEEG findings were unremarkable. In the four remaining patients (28%) findings normalized in follow-up EEGs. Notably, these latter cases reported a marked reduction or even ceasing of attacks.

IEDs were documented for the first time during the follow-up in four further patients (see Table 3).

### Epilepsy suspicion and IEDs

Five patients received a diagnosis of epilepsy prior to admission at our center. Three of them were on antiepileptic therapy (Pt. 3-I valproate, 4-III phenytoin, 6-I phenobarbital) at the time of referral. Eventually, none of the five patients had a convincing history of seizures and diagnosis of epilepsy was not confirmed during the work-up. Antiepileptic drugs were stopped after the diagnosis of FHM1/EA2.

**Fig. 1:**
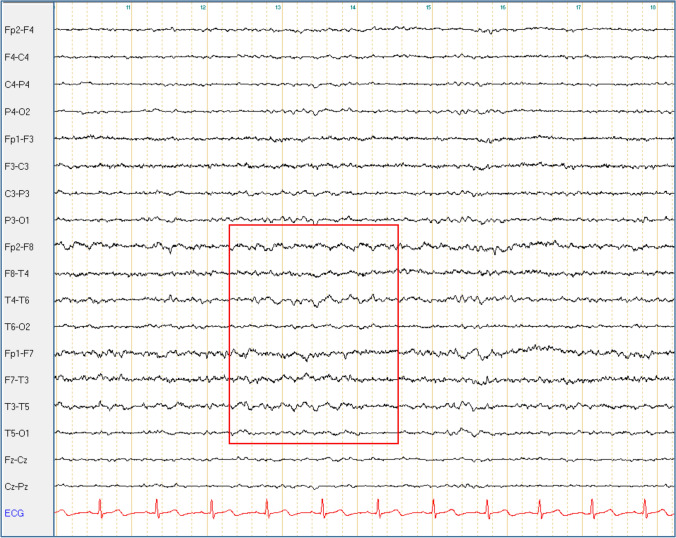
Bitemporal delta activity in Pt. 5-I. Bipolar longitudinal montage with 70 Hz filter and time constant of 0.3 s; sensitivity 7 μV

**Fig. 2:**
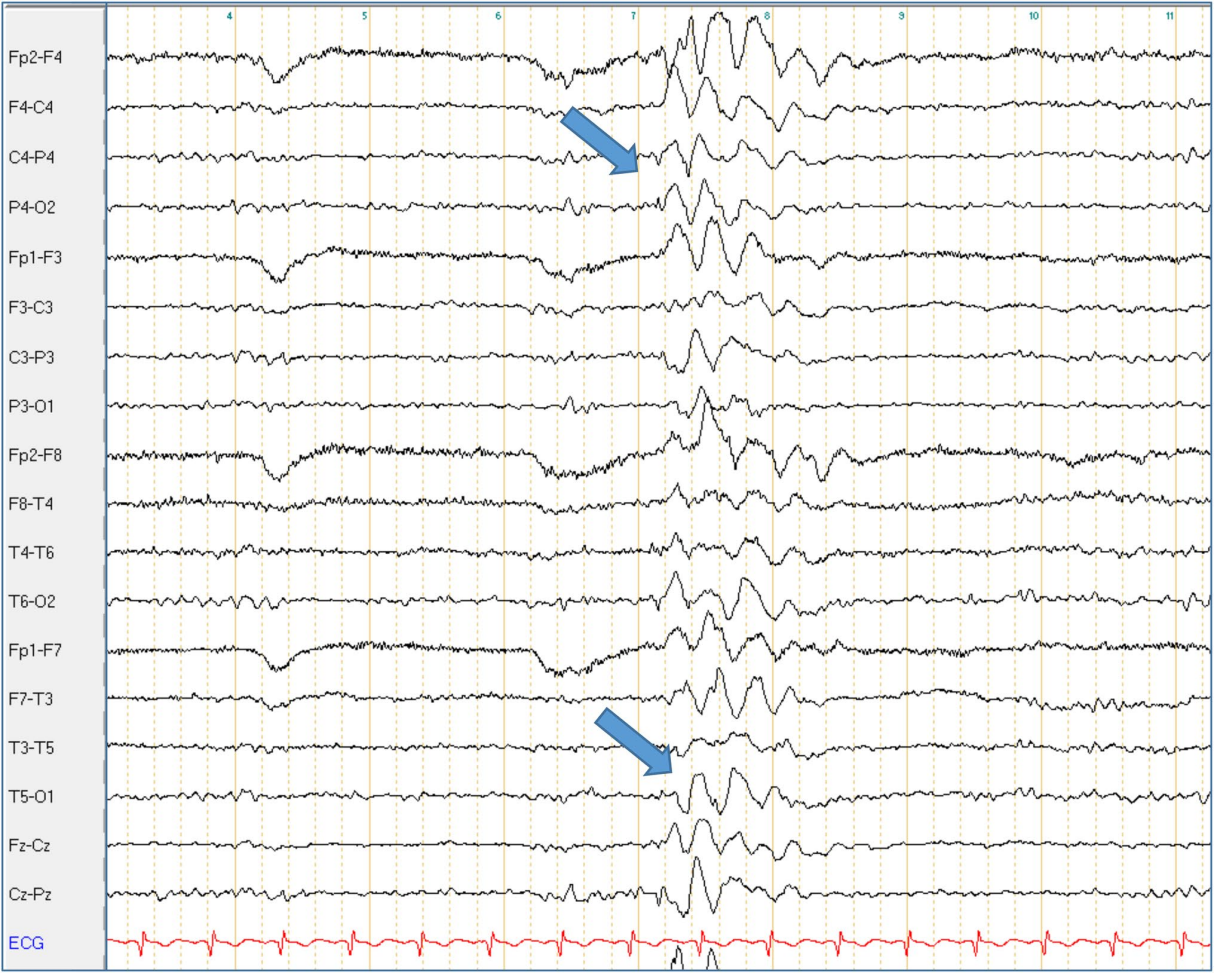
Superimposed bilateral occipital spikes in generalized rhythmic delta activity in Pt. 4-IV. Bipolar longitudinal montage with 70 Hz filter and time constant of 0.3 s; sensitivity 7 μV

**Fig. 3:**
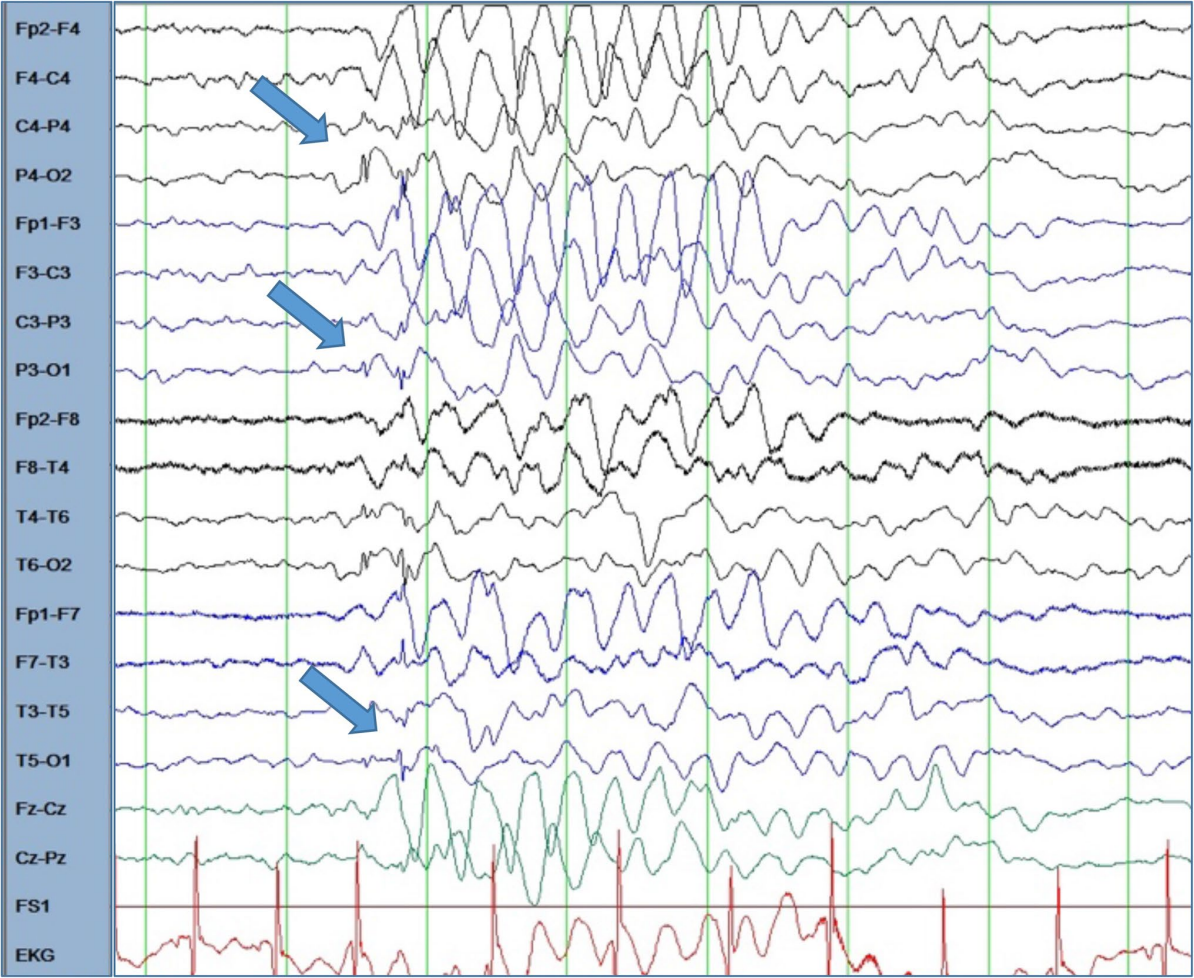
Superimposed bilateral occipital spikes in generalized rhythmic delta activity in Pt. 4-VI. Bipolar longitudinal montage with 70 Hz filter and time constant of 0.3 s; sensitivity 10 μV

**Fig. 4:**
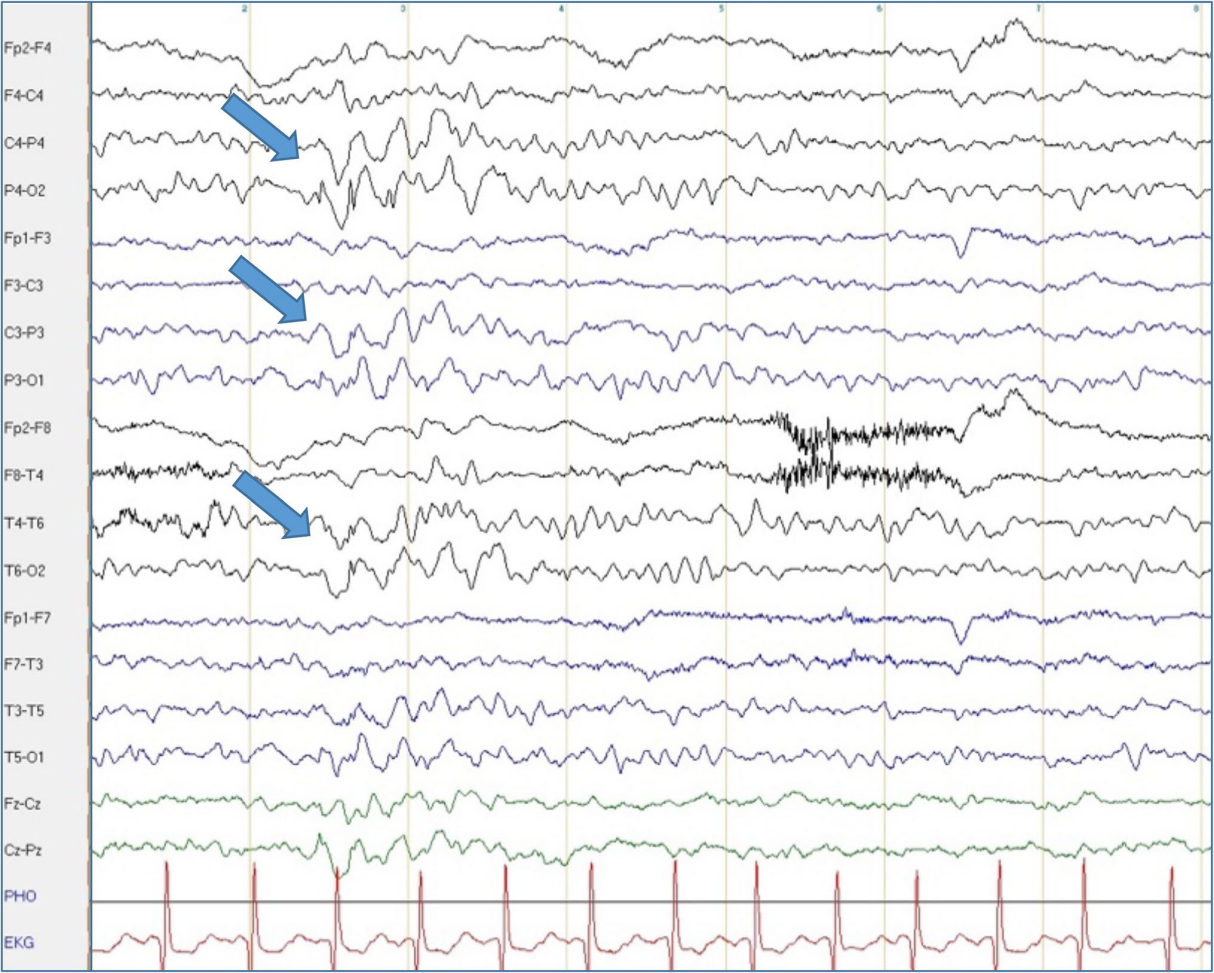
Superimposed bilateral occipital spikes in generalized rhythmic delta activity in Pt. 15-I. Bipolar longitudinal montage with 70 Hz filter and time constant of 0.3 s; sensitivity 10 μV

**Fig. 5:**
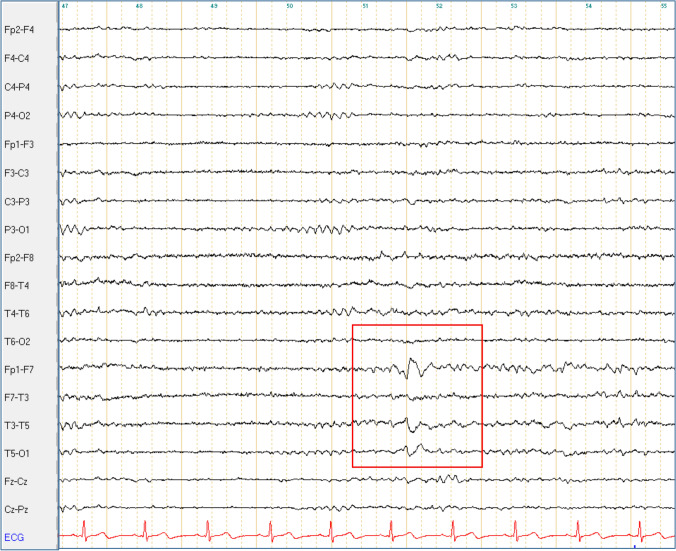
Left temporal sharp waves in Pt. 5-I. Bipolar longitudinal montage with 70 Hz filter and time constant of 0.3 s; sensitivity 7 μV

Conversely, IEDs were detected at different time points in eight patients (21%, 7 EA2 and 1 FHM1, see also Table 3 and Fig. 1). None of them met the diagnostic criteria for epilepsy [12]. In one case (Pt. 15-I), clinical history was suggestive of absences, though no correlating EEG changes were detected in repeated in-patient stays and video-EEG monitoring. Patients with IEDs were significantly younger (16 ± 9 years of age at examination *vs* 43 ± 21 in those without IEDs, *p* = 0.003) and had a significantly lower median age at onset (1(1–2) years *vs* 12(5–45) in those without IEDs, *p* = 0.0009) (Figs. 2, 3, 4, 5).

During the follow-up, a single generalized tonic–clonic seizure was observed during a severe hemiplegic migraine attack in a patient with the S218L mutation (Pt. 6-I). He never displayed epileptic changes in EEG nor had seizures independently from hemiplegic attacks.

### Comparison with *CACNA1A* phenocopies

We retrieved from our database 18 patients with episodic ataxia or sporadic hemiplegic migraine which had been tested negative for *CACNA1A* variants. Fifteen of them underwent EEG examinations at least once and were thus considered for this study (*n* = 10 with EA phenotype, *n* = 3 with sporadic HM, *n* = 2 overlapping phenotype). *CACNA1A*-negative phenocopies were significantly older than *CACNA1A* patients (median age at EEG 59 (29; 67) years in phenocopies *vs* 39 (12; 51) in *CACNA1A* patients, *p* = 0.008).

In all but four cases, who displayed intermittent bilateral temporal slowing (3 cases theta, 1 case delta), EEG findings were unremarkable. Hence, in the comparison with the *CACNA1A* cohort, *CACNA1A*-negative phenocopies had a significantly lower frequency of abnormal findings (*p* = 0.03).

### Review of the literature

The literature search yielded 57 pertinent reports. Notably, available EEG findings were mostly limited to the description of changes occurring during attacks or documentation of IEDs.

Concerning FHM1, several reports described EEG findings during attacks consisting mostly of severe slowing contralateral to hemiparesis [13–22]. Fewer descriptions of EEG findings beyond the attacks are available. These were reported to be normal in several cases [21, 23–27]. In single case, a residual unspecific focal slowing in the hemisphere contralateral to aura symptoms has been reported [13, 14]. Seizures concomitant to severe hemiplegic attacks have been described in at least 16 families [5, 13–17, 19, 20, 22, 24, 27–34]. Seven of these families (44%) harbored the genotype S218L, which is associated with severe attacks with coma—or even death—induced by minor head trauma [14, 16, 19, 29, 31, 33, 34]. Other genotypes recurrently associated with a seizure during attacks were R1349Q (*n* = 3, 19%) [20, 27, 32] and Y1385C (*n* = 2, 12%) [5, 22]. Epilepsy or seizures occurring independently from hemiplegic migraine have been described in at least eight pedigrees [15, 24, 28, 30, 35]. In only two of these cases IEDs were documented [15, 35].

Concerning EA2, numerous reports described IEDs [36–42]. Very few cases reported additional information on EEG findings, which were reported to be normal [43] or with interposed slowing[36, 40]. Descriptions of EEG findings during an attack are lacking. In a report by Labrum et al. EEG during an attack did not differ from baseline EEG in a patient with EA2[40]. In at least 21 EA2 families epilepsy/seizures occurred independently from episodic ataxia [4, 20, 36–41, 44–51]. In virtually all cases, epilepsy manifested in the childhood and preceded episodic ataxia. Absences represented the most common manifestations (*n* = 12, 57%) [20, 36–41, 48, 50, 51]. In 10 out of these 21 families, IEDs could be detected [36–41]. Notably, IEDs were also detected in several other EA2 families with no history of seizures [41, 42, 52, 53]. Generalized 3 Hz spike-wave discharges were a recurrent EEG pattern [37, 39, 41, 54, 55].

## Discussion

In the present work, we systematically analyzed repeated rsEEG recordings of a large cohort of genetically confirmed *CACNA1A* patients, which were collected during a long-term clinical follow-up. In our *CACNA1A* cohort, 55% of the baseline EEGs showed abnormal findings. The key pathological findings consisted of a variable slowing, preferentially affecting the temporal lobe. Generally, slowing pattern was more pronounced following recent attacks. A normalization of EEG findings was mostly observed in association with clinical remission of episodic symptoms. However, during follow-up intermittent lateralized slowing was present in a majority of patients. IEDs were recorded in 21% of patients. Overall, earlier onset of disease was associated with EEG abnormalities, especially IEDs. None of our patients met the diagnostic criteria of epilepsy. Looking at the genotype, all SCA6 patients had normal EEG findings, while EA2 and FHM1 patients had a similar frequency of abnormal findings.

Historically, EEG has been applied in the differential diagnosis of “acetazolamide-responsive periodic ataxia” from epilepsy before a genetic allocation was possible [56]. After the identification of *CACNA1A* mutations, a growing body of literature focused on the co-occurrence of epileptic disorders in FHM1 and EA2 patients. Though, most of the evidence of an association between *CACNA1A* disorders and epilepsy originated from small case series. Systematic electrophysiological studies in larger collectives have been not carried out to date. By analyzing a genetically assigned large single-center cohort, we were able to detect an EEG pattern across two *CACNA1A* genotypes, consisting of a recurrent lateralized EEG slowing in the interval between the attacks. Even if non-specific, this EEG pattern discriminated *CACNA1A* patients from *CACNA1A*-negative phenocopies in our study. We hypothesize that the chronic EEG abnormalities in *CACNA1A* patients primarily reflect a functional disruption of cerebro-cerebellar connections. The occurrence of such network disturbances has been theorized upon observation that FHM1 and EA2 patients frequently display cognitive impairment, developmental delay and behavioral disorders (6). Indeed, cerebellar tuning extends beyond motor control to cognitive and emotional processing through multiple connections with associative areas, especially prefrontal and temporal (58). To date, powerful structural correlates of functional disruption of cerebro-cerebellar pathways are lacking. Indeed, supratentorial structures appear to be spared at conventional brain imaging [57]. Therefore, our findings strengthen the pivotal role of electrophysiology in the clinical workup of *CACNA1A* disorders, even in the interval between attacks.

IEDs were detected in seven EA2 and one FHM1 patients at different time points. All but one were children or young adults with EA2. Interestingly, IEDs in EA2 were recorded without the evidence of a clinical attack during video-monitoring in our cohort and were not recorded in the patients that underwent long-term follow-up and were beyond their mid-20s. These findings are similar to evidence collected in the literature, which recurrently reported IEDs and or seizures in EA2 during childhood, sometimes before the development of episodic ataxia and chronic cerebellar signs [40, 44–46, 50, 51]. Generalized epileptic seizures may also occur in FHM1, as in one of our patients. A recurrent association between severe migraine attacks with prolonged aura accompanied by hemiplegia, aphasia or coma was associated with specific genotypes such as the S218L mutation (see also patient 6-I) [14, 16, 19, 34]. Concomitant and even occasionally fatal brain edema seen in these so-called “malignant” FHM1 mutations imply that these seizures are symptomatic [29]. Interestingly, de novo *CACNA1A* mutations have been recently associated with a sporadic early infantile epileptic encephalopathy (the so-called EIEE42) resulting in a severe clinical phenotype with developmental delay and epilepsy (2). One of our patients (6-I) displayed as child such a severe phenotype that could be classified as EIEE42 within the spectrum of *CACNA1A* mutations. If EIEE42 has to be considered a separate “epileptic” entity or rather an age-dependent manifestation of *CACNA1A* channelopathy will be part of the future studies.

It is widely established that P/Q channels are ubiquitary but unequivocally distributed in the brain. In supratentorial regions, there is especially evidence of their expression in the hippocampus, where their alteration in human specimens and in animal models has been associated to epileptogenesis [58–60], as well as in thalamic neurons [61]. Pivotal studies in murine models showed that thalamic mediated arousal upon stimuli is coupled by the activation of P/Q channels [61]. The depolarization of P/Q channels in the thalamic neurons promotes high-frequency membrane oscillations which facilitate synaptic inputs and the transition in that low-voltage fast activity, the so-called gamma waves, typical of the attentive state and engagement in cognitive tasks [62]. In mice lacking P/Q channel, a residual excitatory transmission between thalamus and cortex is detected, but it is insufficient to maintain high-frequency oscillations [61]. Furthermore, the P/Q-channel deficient mice display bursts of spike and wave activity, resembling an absence status. This finding supports a selective susceptibility for IEDs in the presence of loss-of-function of P/Q channels. Although a propagation of epileptic activity from subcortical structures to the cortex in humans is unusual, impaired P/Q function in *CACNA1A* disorders may result in such a phenomenon. Both, the presence of such propagation and remission of IEDs with aging and maturation of the brain as pathophysiological substrate for EEG abnormalities in our cohort remain speculative.

Cumulatively, our findings show that in patients with episodic ataxia and hemiplegic migraine EEG slowing between the attacks and intermittent IEDs during childhood and adolescence are indicative—together with a positive family history—of a *CACNA1A* mutation. While in the presence of a positive family history diagnosis is straightforward, in sporadic or late onset cases with non-informative family history, detection of EEG abnormalities may support the choice of a genetic screening. Also, persistence of slowing in the weeks after attacks appears useful to discriminate *CACNA1A*-related hemiplegic migraine.

The pathophysiological background of the EEG abnormalities in the setting of P/Q dysfunction in humans remains elusive. Anyhow, the present findings support the increasing evidence that functional pathologic changes expand beyond the cerebellar circuitry in non-polyglutamine *CACNA1A* diseases and strongly suggest an age-dependent effect of P/Q dysfunction.

Future studies are advocated to gain further knowledge on the functional changes related to *CACNA1A* variants in vivo as well as to shed light on the clinical course of early-onset phenotypes.

## Data Availability

The dataset analyzed in the current study is available upon request. All newly reported *CACNA1A* mutations have been submitted to ClinVar at the time of their detection.

## References

[1] Rajakulendran S, Kaski D, Hanna MG (2012). Neuronal P/Q-type calcium channel dysfunction in inherited disorders of the CNS. Nat Rev Neurol.

[2] Epi KC (2016). De Novo Mutations in SLC1A2 and CACNA1A are important causes of epileptic encephalopathies. Am J Hum Genet.

[3] Ophoff RA, Terwindt GM, Vergouwe MN, van Eijk R, Oefner PJ, Hoffman SM (1996). Familial hemiplegic migraine and episodic ataxia type-2 are caused by mutations in the Ca2+ channel gene CACNL1A4. Cell.

[4] Jen J, Kim GW, Baloh RW (2004). Clinical spectrum of episodic ataxia type 2. Neurology.

[5] Ducros A, Denier C, Joutel A, Cecillon M, Lescoat C, Vahedi K (2001). The clinical spectrum of familial hemiplegic migraine associated with mutations in a neuronal calcium channel. N Engl J Med.

[6] Zhuchenko O, Bailey J, Bonnen P, Ashizawa T, Stockton DW, Amos C (1997). Autosomal dominant cerebellar ataxia (SCA6) associated with small polyglutamine expansions in the alpha 1A-voltage-dependent calcium channel. Nat Genet.

[7] Bavassano C, Eigentler A, Stanika R, Obermair GJ, Boesch S, Dechant G (2017). Bicistronic CACNA1A gene expression in neurons derived from spinocerebellar ataxia type 6 patient-induced pluripotent stem cells. Stem Cells Dev.

[8] Jen JC, Yue Q, Karrim J, Nelson SF, Baloh RW (1998). Spinocerebellar ataxia type 6 with positional vertigo and acetazolamide responsive episodic ataxia. J Neurol Neurosurg Psychiatry.

[9] Kane N, Acharya J, Benickzy S, Caboclo L, Finnigan S, Kaplan PW (2017). A revised glossary of terms most commonly used by clinical electroencephalographers and updated proposal for the report format of the EEG findings. Revision 2017. Clin Neurophysiol Pract..

[10] Indelicato E, Nachbauer W, Karner E, Eigentler A, Wagner M, Unterberger I (2019). The neuropsychiatric phenotype in CACNA1A mutations: a retrospective single center study and review of the literature. Eur J Neurol.

[11] Nachbauer W, Nocker M, Karner E, Stankovic I, Unterberger I, Eigentler A (2014). Episodic ataxia type 2: phenotype characteristics of a novel CACNA1A mutation and review of the literature. J Neurol.

[12] Fisher RS, Acevedo C, Arzimanoglou A, Bogacz A, Cross JH, Elger CE (2014). ILAE official report: a practical clinical definition of epilepsy. Epilepsia.

[13] Beauvais K, Cave-Riant F, De Barace C, Tardieu M, Tournier-Lasserve E, Furby A (2004). New CACNA1A gene mutation in a case of familial hemiplegic migraine with status epilepticus. Eur Neurol.

[14] Chan YC, Burgunder JM, Wilder-Smith E, Chew SE, Lam-Mok-Sing KM, Sharma V (2008). Electroencephalographic changes and seizures in familial hemiplegic migraine patients with the CACNA1A gene S218L mutation. J Clin Neurosci.

[15] de Vries B, Stam AH, Beker F, van den Maagdenberg AM, Vanmolkot KR, Laan L (2008). CACNA1A mutation linking hemiplegic migraine and alternating hemiplegia of childhood. Cephalalgia.

[16] Debiais S, Hommet C, Bonnaud I, Barthez MA, Rimbaux S, Riant F (2009). The FHM1 mutation S218L: a severe clinical phenotype? A case report and review of the literature. Cephalalgia.

[17] Garcia Segarra N, Gautschi I, Mittaz-Crettol L, Kallay Zetchi C, Al-Qusairi L, Van Bemmelen MX (2014). Congenital ataxia and hemiplegic migraine with cerebral edema associated with a novel gain of function mutation in the calcium channel CACNA1A. J Neurol Sci.

[18] Ohmura K, Suzuki Y, Saito Y, Wada T, Goto M, Seto S (2012). Sporadic hemiplegic migraine presenting as acute encephalopathy. Brain Dev.

[19] Stam AH, Luijckx GJ, Poll-The BT, Ginjaar IB, Frants RR, Haan J (2009). Early seizures and cerebral oedema after trivial head trauma associated with the CACNA1A S218L mutation. J Neurol Neurosurg Psychiatry.

[20] Tantsis EM, Gill D, Griffiths L, Gupta S, Lawson J, Maksemous N (2016). Eye movement disorders are an early manifestation of CACNA1A mutations in children. Dev Med Child Neurol.

[21] Topakian R, Pischinger B, Stieglbauer K, Pichler R (2014). Rare clinical findings in a patient with sporadic hemiplegic migraine: FDG-PET provides diminished brain metabolism at 10-year follow-up. Cephalalgia.

[22] Vahedi K, Denier C, Ducros A, Bousson V, Levy C, Chabriat H (2000). CACNA1A gene de novo mutation causing hemiplegic migraine, coma, and cerebellar atrophy. Neurology.

[23] Kors EE, Haan J, Giffin NJ, Pazdera L, Schnittger C, Lennox GG (2003). Expanding the phenotypic spectrum of the CACNA1A gene T666M mutation: a description of 5 families with familial hemiplegic migraine. Arch Neurol.

[24] Kors EE, Melberg A, Vanmolkot KR, Kumlien E, Haan J, Raininko R (2004). Childhood epilepsy, familial hemiplegic migraine, cerebellar ataxia, and a new CACNA1A mutation. Neurology.

[25] Blumkin L, Michelson M, Leshinsky-Silver E, Kivity S, Lev D, Lerman-Sagie T (2010). Congenital ataxia, mental retardation, and dyskinesia associated with a novel CACNA1A mutation. J Child Neurol.

[26] Liguori C, Albanese M, Sancesario G, Stefani A, Marciani MG, Pierantozzi M (2013). May a suspicious psychiatric disorder hide sporadic hemiplegic migraine? Genetic test as prompting factor for diagnosis. Neurol Sci.

[27] Sanchez-Albisua I, Schoning M, Jurkat-Rott K, Lerche H (2013). Possible effect of corticoids on hemiplegic attacks in severe hemiplegic migraine. Pediatr Neurol.

[28] Riant F, Ducros A, Ploton C, Barbance C, Depienne C, Tournier-Lasserve E (2010). De novo mutations in ATP1A2 and CACNA1A are frequent in early-onset sporadic hemiplegic migraine. Neurology.

[29] Kors EE, Terwindt GM, Vermeulen FL, Fitzsimons RB, Jardine PE, Heywood P (2001). Delayed cerebral edema and fatal coma after minor head trauma: role of the CACNA1A calcium channel subunit gene and relationship with familial hemiplegic migraine. Ann Neurol.

[30] Stam AH, Vanmolkot KR, Kremer HP, Gartner J, Brown J, Leshinsky-Silver E (2008). CACNA1A R1347Q: a frequent recurrent mutation in hemiplegic migraine. Clin Genet.

[31] Carreno O, Garcia-Silva MT, Garcia-Campos O, Martinez-de Aragon A, Cormand B, Macaya A (2011). Acute striatal necrosis in hemiplegic migraine with de novo CACNA1A mutation. Headache.

[32] Malpas TJ, Riant F, Tournier-Lasserve E, Vahedi K, Neville BG (2010). Sporadic hemiplegic migraine and delayed cerebral oedema after minor head trauma: a novel de novo CACNA1A gene mutation. Dev Med Child Neurol.

[33] Zangaladze A, Asadi-Pooya AA, Ashkenazi A, Sperling MR (2010). Sporadic hemiplegic migraine and epilepsy associated with CACNA1A gene mutation. Epilepsy Behav.

[34] Yamazaki S, Ikeno K, Abe T, Tohyama J, Adachi Y (2011). Hemiconvulsion-hemiplegia-epilepsy syndrome associated with CACNA1A S218L mutation. Pediatr Neurol.

[35] Romaniello R, Zucca C, Tonelli A, Bonato S, Baschirotto C, Zanotta N (2010). A wide spectrum of clinical, neurophysiological and neuroradiological abnormalities in a family with a novel CACNA1A mutation. J Neurol Neurosurg Psychiatry.

[36] Damaj L, Lupien-Meilleur A, Lortie A, Riou E, Ospina LH, Gagnon L (2015). CACNA1A haploinsufficiency causes cognitive impairment, autism and epileptic encephalopathy with mild cerebellar symptoms. Eur J Hum Genet.

[37] Imbrici P, Jaffe SL, Eunson LH, Davies NP, Herd C, Robertson R (2004). Dysfunction of the brain calcium channel CaV2.1 in absence epilepsy and episodic ataxia. Brain.

[38] Jouvenceau A, Eunson LH, Spauschus A, Ramesh V, Zuberi SM, Kullmann DM (2001). Human epilepsy associated with dysfunction of the brain P/Q-type calcium channel. Lancet.

[39] Jung J, Testard H, Tournier-Lasserve E, Riant F, Vallet AE, Berroir S (2010). Phenotypic variability of episodic ataxia type 2 mutations: a family study. Eur Neurol.

[40] Labrum RW, Rajakulendran S, Graves TD, Eunson LH, Bevan R, Sweeney MG (2009). Large scale calcium channel gene rearrangements in episodic ataxia and hemiplegic migraine: implications for diagnostic testing. J Med Genet.

[41] Rajakulendran S, Graves TD, Labrum RW, Kotzadimitriou D, Eunson L, Davis MB (2010). Genetic and functional characterisation of the P/Q calcium channel in episodic ataxia with epilepsy. J Physiol.

[42] Kaido M, Furuta M, Nakamori M, Yuasa Y, Takahashi MP (2016). Episodic ataxia type 2 manifests as epileptiform electroencephalographic activity with no epileptic attacks in two family members. Rinsho Shinkeigaku.

[43] Yugrakh MS, Levy OA (2012). Clinical reasoning: a middle-aged man with episodes of gait imbalance and a newly found genetic mutation. Neurology.

[44] Choi JH, Seo JD, Choi YR, Kim MJ, Shin JH, Kim JS (2015). Exercise-induced downbeat nystagmus in a Korean family with a nonsense mutation in CACNA1A. Neurol Sci.

[45] Choi KD, Yook JW, Kim MJ, Kim HS, Park YE, Kim JS (2013). Possible anticipation associated with a novel splice site mutation in episodic ataxia type 2. Neurol Sci.

[46] Imbrici P, Eunson LH, Graves TD, Bhatia KP, Wadia NH, Kullmann DM (2005). Late-onset episodic ataxia type 2 due to an in-frame insertion in CACNA1A. Neurology.

[47] Kaunisto MA, Harno H, Kallela M, Somer H, Sallinen R, Hamalainen E (2004). Novel splice site CACNA1A mutation causing episodic ataxia type 2. Neurogenetics.

[48] Mantuano E, Romano S, Veneziano L, Gellera C, Castellotti B, Caimi S (2010). Identification of novel and recurrent CACNA1A gene mutations in fifteen patients with episodic ataxia type 2. J Neurol Sci.

[49] Riant F, Lescoat C, Vahedi K, Kaphan E, Toutain A, Soisson T (2010). Identification of CACNA1A large deletions in four patients with episodic ataxia. Neurogenetics.

[50] Strupp M, Kalla R, Dichgans M, Freilinger T, Glasauer S, Brandt T (2004). Treatment of episodic ataxia type 2 with the potassium channel blocker 4-aminopyridine. Neurology.

[51] Stendel C, D'Adamo MC, Wiessner M, Dusl M, Cenciarini M, Belia S, et al. Association of A Novel Splice Site Mutation in P/Q-Type Calcium Channels with Childhood Epilepsy and Late-Onset Slowly Progressive Non-Episodic Cerebellar Ataxia. Int J Mol Sci. 2020;21(11). 10.3390/ijms21113810 PMC7312673 32471306

[52] Baloh RW, Yue Q, Furman JM, Nelson SF (1997). Familial episodic ataxia: clinical heterogeneity in four families linked to chromosome 19p. Ann Neurol.

[53] Cuenca-Leon E, Banchs I, Serra SA, Latorre P, Fernandez-Castillo N, Corominas R (2009). Late-onset episodic ataxia type 2 associated with a novel loss-of-function mutation in the CACNA1A gene. J Neurol Sci.

[54] Du X, Chen Y, Zhao Y, Luo W, Cen Z, Hao W (2017). Dramatic response to pyridoxine in a girl with absence epilepsy with ataxia caused by a de novo CACNA1A mutation. Seizure.

[55] Nardello R, Plicato G, Mangano GD, Gennaro E, Mangano S, Brighina F (2020). Two distinct phenotypes, hemiplegic migraine and episodic Ataxia type 2, caused by a novel common CACNA1A variant. BMC Neurol.

[56] Neufeld MY, Nisipeanu P, Chistik V, Korczyn AD (1996). The electroencephalogram in acetazolamide-responsive periodic ataxia. Mov Disord.

[57] Russell MB, Ducros A (2011). Sporadic and familial hemiplegic migraine: pathophysiological mechanisms, clinical characteristics, diagnosis, and management. Lancet Neurol.

[58] Djamshidian A, Grassl R, Seltenhammer M, Czech T, Baumgartner C, Schmidbauer M (2002). Altered expression of voltage-dependent calcium channel alpha(1) subunits in temporal lobe epilepsy with Ammon's horn sclerosis. Neuroscience.

[59] Xu JH, Tang FR. Voltage-dependent calcium channels, calcium binding proteins, and their interaction in the pathological process of epilepsy. Int J Mol Sci. 2018;19(9). 10.3390/ijms19092735 PMC6164075 30213136

[60] Hell JW, Westenbroek RE, Warner C, Ahlijanian MK, Prystay W, Gilbert MM (1993). Identification and differential subcellular localization of the neuronal class C and class D L-type calcium channel alpha 1 subunits. J Cell Biol.

[61] Llinas RR, Choi S, Urbano FJ, Shin HS (2007). Gamma-band deficiency and abnormal thalamocortical activity in P/Q-type channel mutant mice. Proc Natl Acad Sci USA.

[62] Pedroarena C, Llinas R (1997). Dendritic calcium conductances generate high-frequency oscillation in thalamocortical neurons. Proc Natl Acad Sci USA.

